# Cultivation factors affecting durum wheat performance under speed breeding conditions

**DOI:** 10.3389/fpls.2025.1630915

**Published:** 2025-08-18

**Authors:** Merve Bayhan, Remzi Özkan, Önder Albayrak, Cuma Akıncı, Mehmet Yıldırım

**Affiliations:** ^1^ Department of Field Crops, Faculty of Agriculture, Dicle University, Diyarbakir, Türkiye; ^2^ Department of Crop and Livestock Production, Hakkari University, Hakkari, Türkiye; ^3^ Department of Field Crops, Faculty of Agriculture, Recep Tayyip Erdoğan University, Rize, Türkiye

**Keywords:** speed breeding, wheat, fertilizer, soil type, pot size

## Abstract

**Introduction:**

This study examined the effects of pot size, soil type, fertilizer x dose interactions, and foliar fertilizer application on wheat growth under speed breeding conditions.

**Methods:**

The study was conducted in 2020 in a semi-controlled greenhouse at Dicle University, Diyarbakır, Türkiye, with a 22-hour photoperiod, 22/17°C day/ night temperature, 70% humidity, and 316.15 µmol/m^2^/s light intensity using a mix of white, red, yellow, and purple LED lamps. Sampiyon durum wheat cultivar was used as plant material.

**Results:**

The highest productivity was achieved in a deep pot (270 cm^3^), 100% peat soil, and 20.20.0 (%20 N + %20 K) fertilizer application. Among the foliar fertilizer applications, the best results were observed in the tillering stage and stem elongation-heading stages.

**Discussion:**

These findings highlight ways to optimize plant productivity and improve the efficiency of the speed breeding process. Because the primary goal of speed breeding is to obtain multiple generations in a short period, determining suitable conditions will contribute to the rapid development of more resilient and productive plant varieties in the future.

## 1 Introduction

Global population growth continues to increase demand for higher-yielding plants (Li et al., 2018). By 2050, the world will need to grow 60–80% more food to feed its population (Tilman et al., 2011; Pardey et al., 2014). Meeting this growing demand will require more efficient crop improvement strategies.

Conventional breeding has successfully developed high-yielding, pest- and stress-tolerant cultivars by crossing parent lines and selecting desirable traits over multiple generations. Common methods include mass selection, pure-line selection, hybridization, and backcrossing, depending on crop pollination type. However, these approaches are time-consuming and limit genetic gain, prompting breeders to seek faster and more cost-effective alternatives (Lamichhane and Thapa, 2022; Singh and Janeja, 2021). One of these is speed breeding (SB), which is an important new technique in modern plant breeding. SB is a modern technique designed to shorten the breeding cycle and accelerate crop improvement by rapidly advancing generations under controlled conditions. By optimizing light, temperature, and other growth factors, SB promotes faster growth and earlier flowering. This allows breeders to develop new crop varieties more quickly, leading to faster genetic gains and improved yields (Watson et al., 2018; Ghosh et al., 2018).

Achieving genetic gain—defined as the improvement in the average performance of progeny over successive selection cycles—is often constrained by the long generation times in conventional breeding. This gain is influenced by factors such as genetic variability, heritability, selection intensity, and breeding duration. Although the use of advanced molecular and omics tools can help recover lost genetic variation, and high-throughput genotyping and phenotyping technologies can enhance selection efficiency (Ratnakumar and Pandey, 2020), reducing the overall time required for breeding remains a key factor in maximizing genetic progress (Xu et al., 2017). In this context, SB offers a significant advantage by accelerating trait introgression and enabling the rapid development of homozygous lines. This parallel advancement of trait selection and line development contributes to greater genetic gain in a shorter time frame (Gudi et al., 2022).

SB relies on precisely controlled environments that promote rapid plant growth, flowering, and seed production. Growth chambers, greenhouses, or specialized rooms are used to regulate temperature, humidity, and light. A key aspect of SB is manipulating photoperiods—adjusting light and dark cycles to meet the needs of specific crops. For example, long-day crops like chickpea and wheat flower earlier under extended light periods, typically achieved with supplemental lighting (Ghosh et al., 2018). This accelerates development, increases biomass, and improves traits such as stem digestibility in crops like switchgrass (Zhao et al., 2017). Extended photoperiods are widely used not only to shorten generation time but also to enhance phenotyping and genetic transformation processes (Ghosh et al., 2018). Although SB has been more challenging for short-day crops due to their specific flowering cues, recent studies by Lee et al. (2023) have developed SB protocols for crops such as sorghum and pigeonpea.

Australian researchers have reported that this innovation could increase wheat production by up to three times. In addition, they found that up to six generations of spring wheat and barley could be grown each year using controlled temperatures and extended photoperiod. This would allow hybrid lines to develop more quickly (Watson et al., 2018). Cha et al (2023) also reported that, in addition to SB, a rapid vernalisation system was being developed that would allow up to five generations of wheat and barley to be grown annually at higher vernalisation temperatures.

In SB conditions, increasing plant density is crucial for improving efficiency. In crops, such as wheat and barley, dense planting accelerates flowering. This higher density creates stress by increasing competition among plants, which promotes early flowering (Takeno, 2016; Ghosh et al., 2018). However, due to early flowering, the total leaf area, biomass, and grain number per spike usually decrease (Bhatta et al., 2021), leading to a reduction in grain yield (Watson et al., 2018).

Owing to the accelerated growth of plants, it is necessary to address fertilization and nutrient deficiencies. Regularly supplying essential nutrients based on pot size and soil type is crucial. To prevent early leaf yellowing and reduce plant vitality and seed formation, it is recommended to apply plant nutrients every one or two weeks. Additionally, under speed conditions, foliar fertilizers can be used to prevent nutrient deficiency and leaf damage. Issues such as calcium deficiency, which often arise during the early growth stages, can be resolved through the use of foliar fertilizers (Alahmad et al., 2018; Ghosh et al., 2018; Bayhan et al., 2022).

To increase grain yield and reduce input costs, simplifying practices and automating processes, such as water and fertilizer application, are crucial in SB conditions. This study focuses on optimizing cultivation techniques under SB conditions by examining the effects of factors such as pot size, soil type, nutrients, and foliar fertilization on wheat growth, distinguishing it from previous studies. While Alahmad et al. (2018) concentrated on developing a multitrait phenotyping method for early-generation selection, Ghosh et al. (2018) addressed the general setup and scalability of SB environments, and Bayhan et al. (2022) evaluated the impact of input levels on generation time and agronomic traits, the present study provides a practical contribution by directly improving the environmental and technical conditions necessary for effective SB implementation.

## 2 Materials and methods

### 2.1 Research site and plant material

This study was conducted in 2020 in a semi-controlled greenhouse at the Faculty of Agriculture, Dicle University, Diyarbakır, Turkey (37°53’ N, 40°16’ E). The greenhouse was equipped with plant-growing tables, air conditioning, water and electricity sources, and a humidifier to ensure optimal growth conditions for the plants. In this study, the Sampiyon durum wheat cultivar, developed by the Faculty of Agriculture at Dicle University and previously shown to perform well under SB conditions (Bayhan et al., 2024; Ozkan et al., 2025), was used as plant material. This cultivar is a drought-tolerant, medium-early maturing genotype with high yield and quality.

### 2.2 Speed breeding conditions

**Figure 1:**
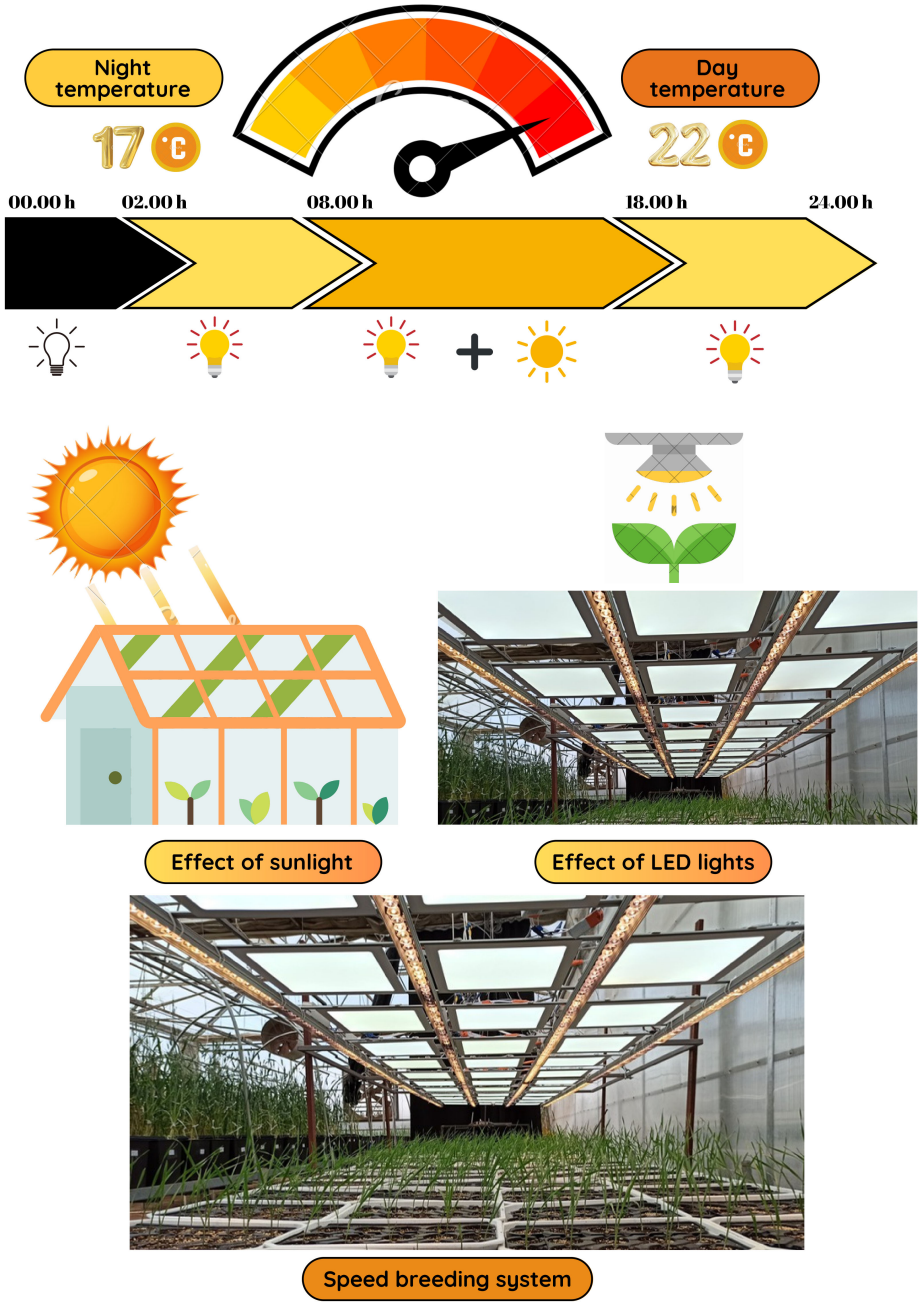
Speed breeding conditions and optimization.

The experimental setup followed the speed breeding protocol outlined by Ghosh et al. (2018), which enabled rapid generation turnover through extended photoperiods and optimized growth conditions. The semi-controlled greenhouse was maintained at 22°C/17°C (day/night) with 70% humidity and a 22-hour light/2-hour dark photoperiod to promote optimal plant growth. Illumination was provided by a mix of white, red, yellow, and purple LED lamps, delivering a light intensity of 316.15 μmol/m²/s at a height (20 cm). Environmental data were monitored using a Trotec BL30 Data Logger, and irrigation was managed using a timer-controlled sprinkler system with solenoid valves (**Figure 1**).

### 2.3 Experiments

**Figure 2:**
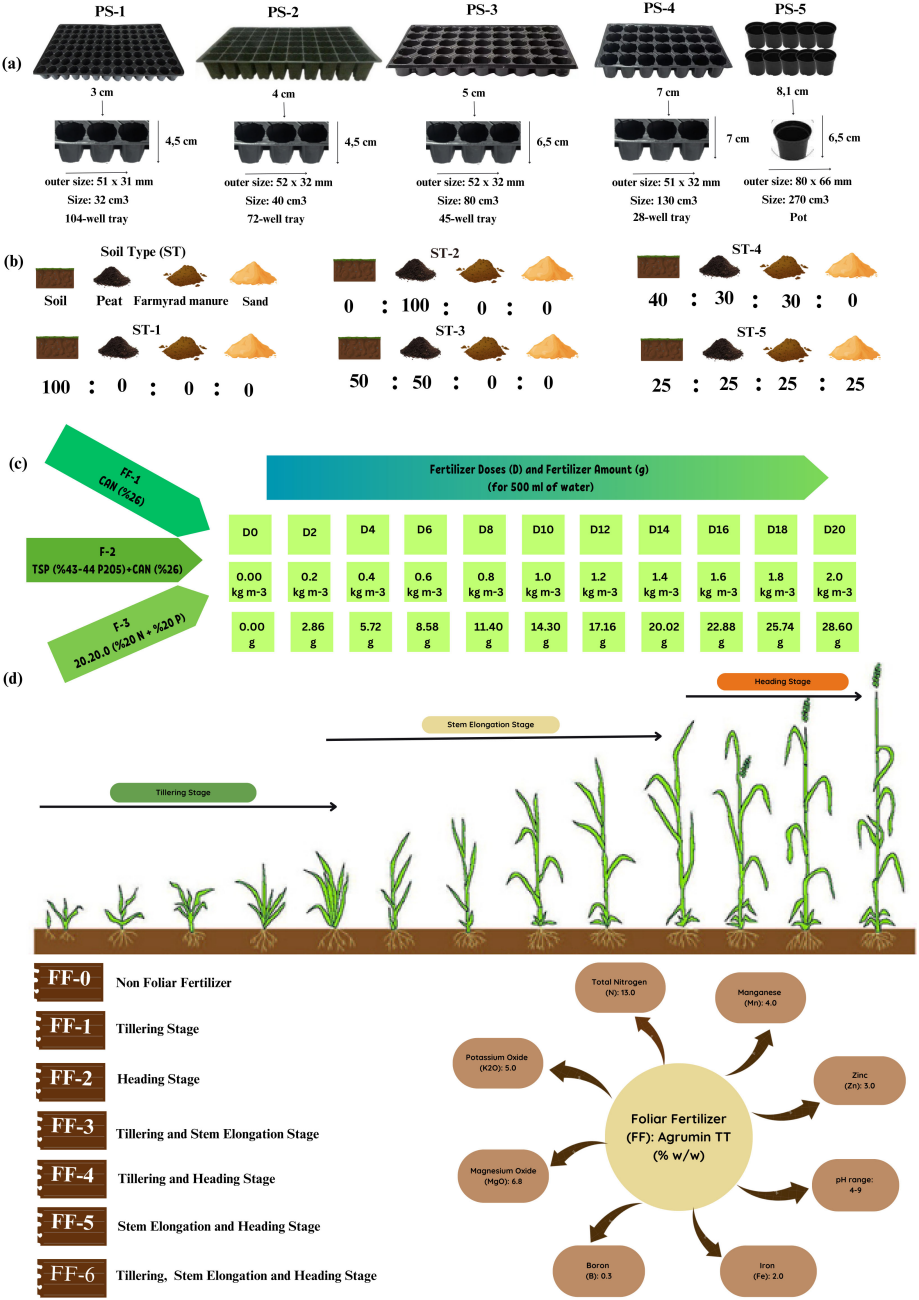
Optimization of different treatments under SB conditions **(a)** different pot sizes, **(b)** different soil types and mix combinations, **(c)** different fertilizer types and doses, **(d)** foliar fertilizer application, PS, Pot size; ST, Soil type; F, Fertilizer; FF, Foliar fertilizer.

Four independent experiments were conducted under SB conditions to optimize the key cultivation parameters. Each experiment focused on a specific variable: pot size (**Figure 2a**), soil type and composition (**Figure 2b**), fertilizer type and dose (**Figure 2c**), and timing of foliar fertilizer application (**Figure 2d**).

#### 2.3.1 Determination of pot size

To determine the optimal pot size for plant development under SB conditions, different pot types were tested as illustrated in **Figure 2a**. The experimental design included seedling trays with varying cell volumes: 104-cell trays (32 cm³), 72-cell trays (40 cm³), 45-cell trays (80 cm³), and 28-cell trays (130 cm³), in addition to single pots with a volume of 270 cm³.

#### 2.3.2 Optimization of soil material

As part of this study, an experiment was conducted to identify the most suitable soil composition for SB conditions. As shown in **Figure 2b**, the treatments included soil, peat, farmyard manure, and sand, as well as various combinations of these materials.

#### 2.3.3 Fertilizer application

To identify the most suitable fertilizer type and optimal application dose under SB conditions, a factorial experiment was conducted using three fertilizer types: F1: CAN (26%), F2: TSP (43–44%) + CAN (26%), and F3: composite fertilizer (20-20-0)—combined with eleven dose levels (D0 = 0 kg m^-^³, D2 = 0.2 kg m^-^³, D4 = 0.4 kg m^-^³, D6 = 0.6 kg m^-^³, D8 = 0.8 kg m^-^³, D10 = 1.0 kg m^-^³, D12 = 1.2 kg m^-^³, D14 = 1.4 kg m^-^³, D16 = 1.6 kg m^-^³, D18 = 1.8 kg m^-^³, D20 = 2.0 kg m^-^³). As shown in **Figure 2c**, all treatments were applied at three key growth stages: sowing, tillering, and spiking. Granular fertilizers were dissolved in water and applied as liquid solutions.

#### 2.3.4 Effect of foliar fertilizer

As illustrated in **Figures 2a, d** foliar fertilization experiment was conducted to evaluate the impact of application timing on different wheat growth stages. The treatments included a control (no foliar application) and six stages: tillering, heading, tillering–stem elongation, tillering–heading, stem elongation–heading, and tillering–stem elongation–heading. Agrumin TT (13-0-5 (6.8 MgO) + ME) was applied at a concentration of 1 kg m^-^³.

### 2.4 Experiment design

**Figure 3:**
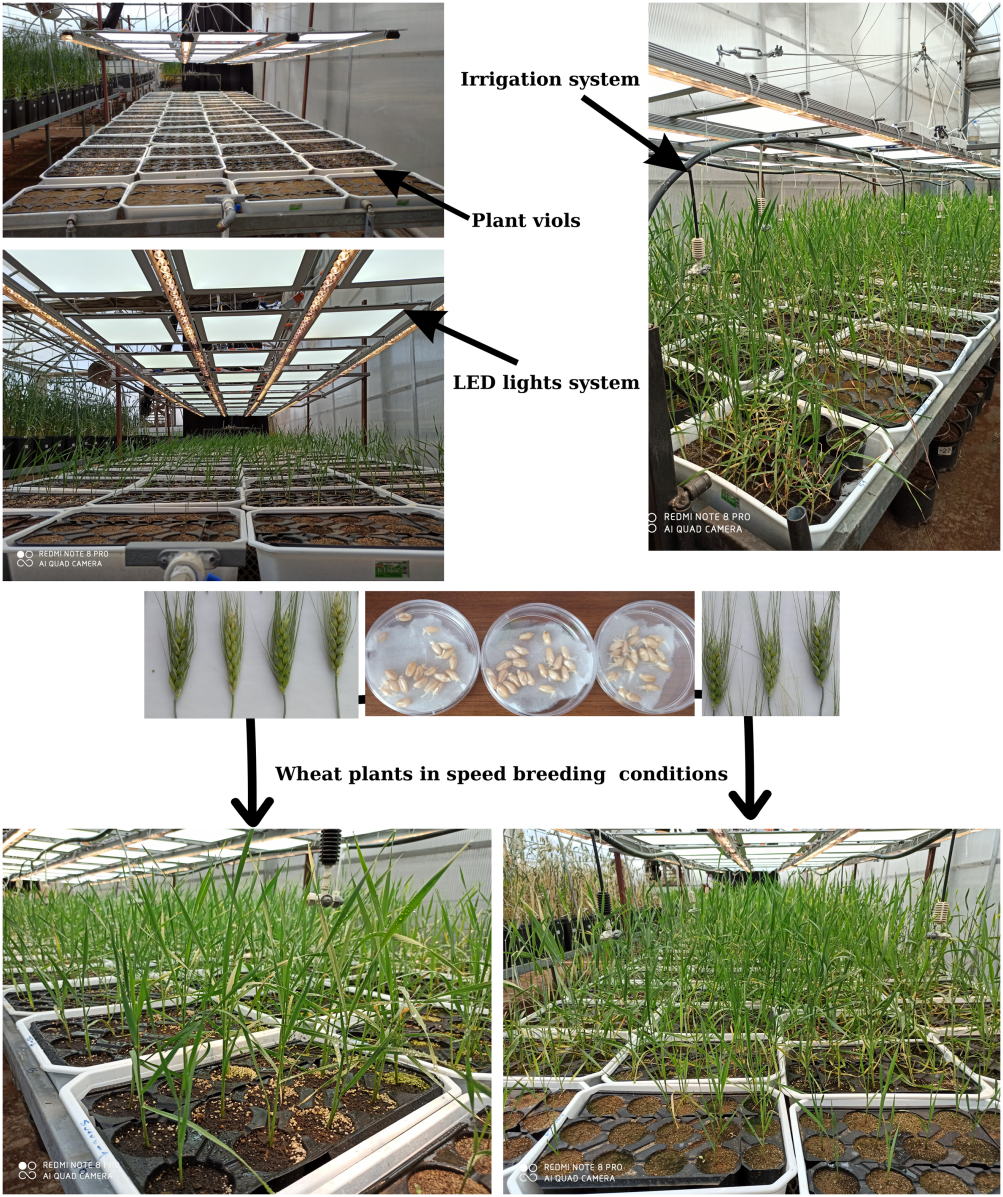
Speed breeding conditions and experimental design.

**Figure 4:**
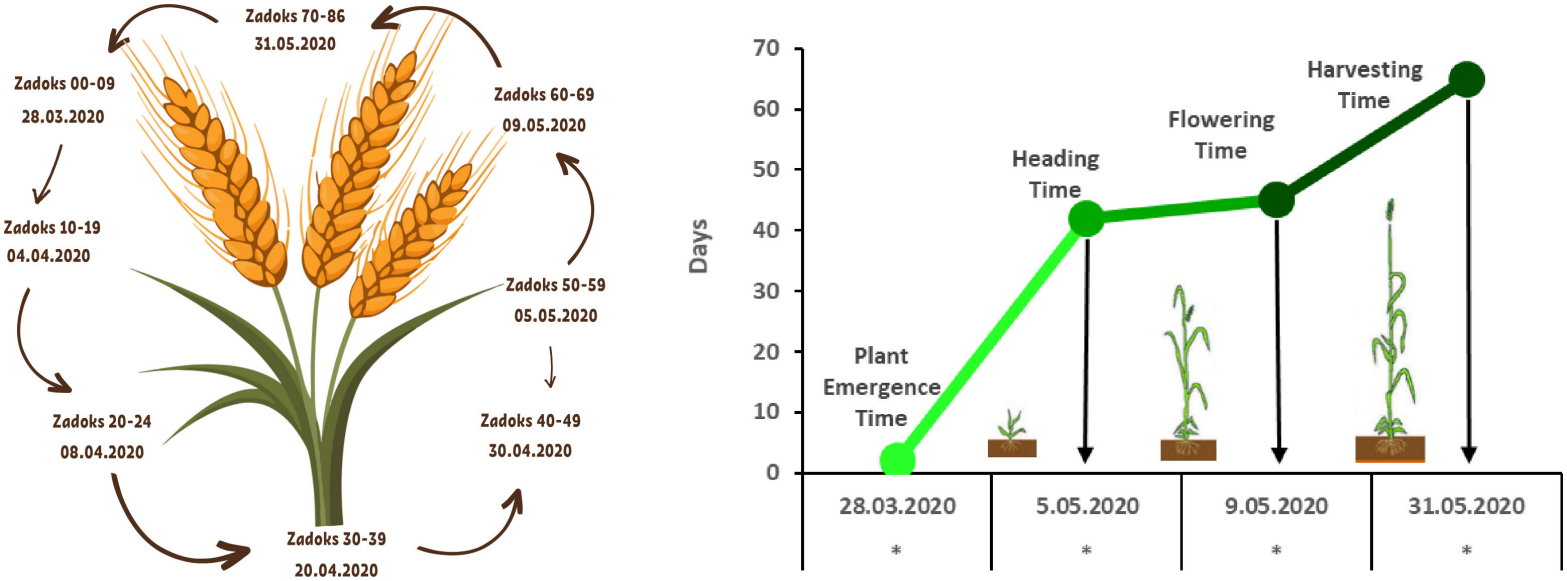
Growth and development periods of wheat under speed breeding conditions (Zadoks et al., 1974).

Except for the pot size experiment, seeds were sown in 28-cell seedling trays, each with a cell volume of 130 cm³. Each tray was considered a replicate, and all experiments were conducted with four replications using a Randomised Plots Experimental Design (Montgomery, 2017). As illustrated in **Figure 3**, one seed was planted per cell, and commercially available peat and farmyard was used as the growth medium (**Table 1**). Sowing was performed on March 25, 2020. With the exception of the fertilizer application experiment, all treatments received calcium ammonium nitrate (CAN, 26%) and a compound fertilizer (20-20-0) at a dose of 1 kg m^-^³ during the sowing, tillering, and heading stages. Irrigation was applied regularly from sowing to heading and then gradually reduced. Plants were harvested 20 days after anthesis. Growth and developmental stages were recorded in days according to the Zadoks scale, as shown in **Figure 4** (Zadoks et al., 1974).

**Table 1:** Physical and chemical properties of the soil, peat and farmyard used in the study.

Analysis name	
Soil	Results
Saturation (%)	63
Salinity (dS/m)	0.92
pH	8.11
Organic Matter (%)	0.71
Nitrogen (%)	0.04
Phosphorus (ppm)	4
Potassium (ppm)	314.45
Calcium (ppm)	10717.89
Magnesium (ppm)	471.78
Sodium (ppm)	26.65
Iron (ppm)	9.29
Copper (ppm)	1.61
Manganese (ppm)	16.5
Zinc (ppm)	0.08
Peat	Results
pH (CaCl_2_)	4.2 – 5.2
pH (H_2_O)	5.0 – 6.0
Electrical Conductivity (EC, µS/cm)	37.2 – 96.7
Lime Content (%)	0 – 0.2
Water Holding Capacity (%)	1126 – 1385
Organic Matter (%)	88 – 95
Farmyard	Results
Total Nitrogen (%)	3.82
Organic Content (%)	61.59

### 2.5 Investigated parameters

In each experiment, a random selection of ten plants was made to assess key morphological and yield-related traits. The parameters measured included plant height (cm), spike length (cm), the number of spikelets per spike, the number of grains per spike, and grain weight (g). These measurements were conducted in accordance with the protocol outlined by Watson et al. (2018).

### 2.6 Statistics analysis

The experimental data were subjected to statistical analysis utilizing JMP 13 statistical software. An analysis of variance (ANOVA) was conducted for each measured trait to evaluate the effects of various treatments. Treatment means were compared using the LSD test for significant differences (p < 0.05).

## 3 Results and discussion

**Figure 5:**
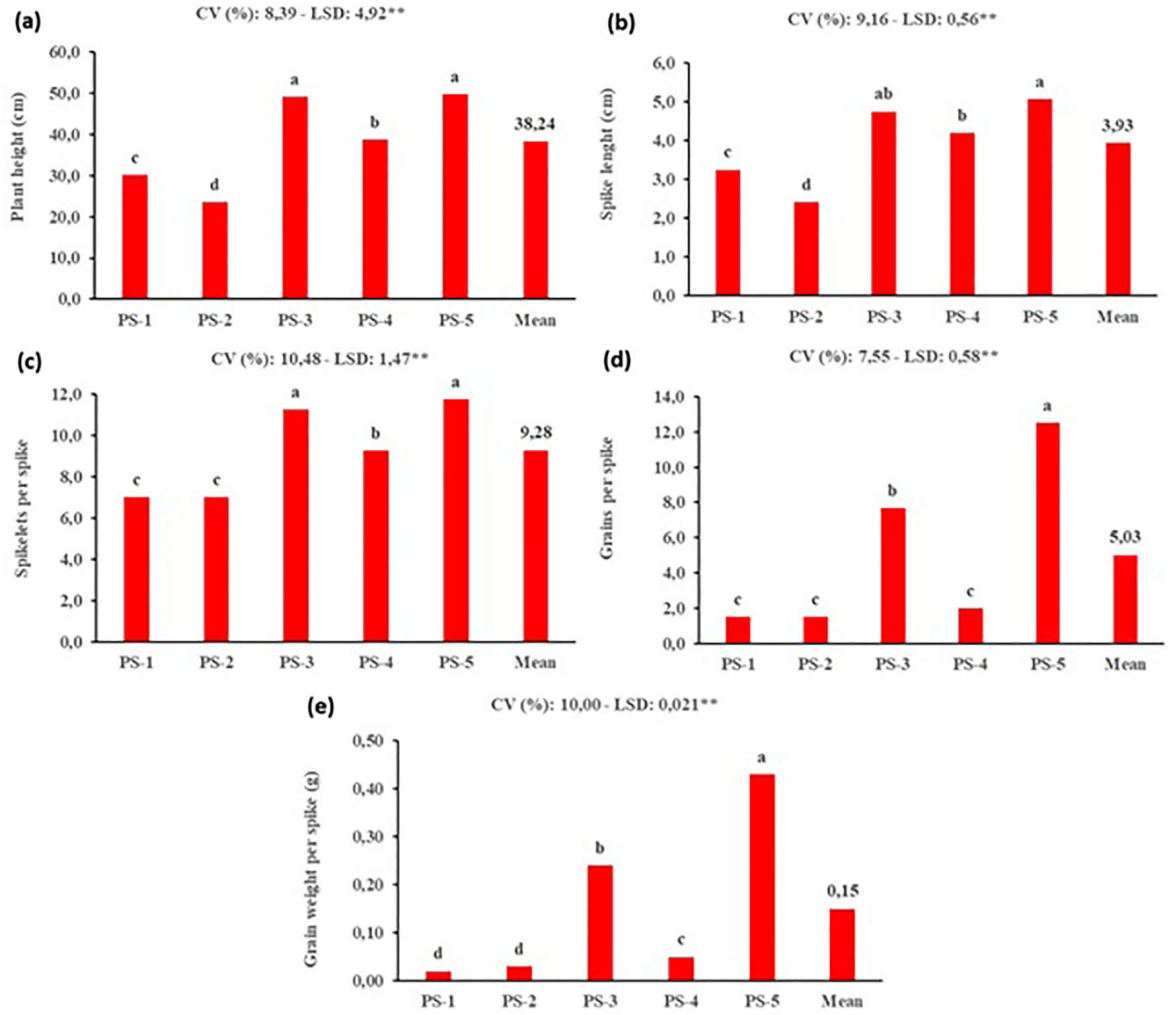
Effects of different pot sizes on yield and yield components of durum wheat under speed breeding condition (PS, Pot size, PS-1: 32 cm^3–^104 hole tray, PS-2: 40 cm^3–^72 hole tray, PS-^3^: 80 cm^3–^45 hole tray, PS-4: 130 cm^3–^28 hole tray, PS-5: 270 cm^3^ pot, CV, Coefficient of variation; LSD, Least significant difference). **(a)** plant height (cm), **(b)** spike lenght (cm), **(c)** spikelet per spike, **(d)** grains per spike and **(e)** grain weight per spike (g). The bar was presented as the standard deviation of means. Different lowercase letters indicated significant differences according to LSD’s test at the level of P<0.01.

The highest values for the characteristics examined in this study were observed for PS-5, which had the deepest pot. As the root spread size decreased, the plant productivity declined (**Figure 5**). Deeper pots allow wheat roots to penetrate deeper into the soil, increasing their ability to seek water and nutrients more widely. 270 cm³ pots with a surface area of 50 cm² can evaluate up to 20,000 plants in a greenhouse with a net useable space of 100 m². Approximately 355 g of soil would be contained in each 270 cm³ container if the average density of the soil combination was 1.3 g/cm³. The same 100 m² greenhouse would require approximately 7.1 kilograms of soil in this scenario. This volume and weight are seen as a feasible and practical scale, consistent with the high-throughput aims of speed breeding programs. Optimizing conditions may increase initial or operational costs, but the reduction in breeding cycle time compared to conventional breeding for 20,000 plants (5–6 years and 1 decare) boosts economic feasibility. Speed breeding’s fast production and market release of high-value novel varieties more than offsets these input costs, making it economically viable for breeders.

In this study, conducted under SB conditions, the influence of pot depth on wheat plant development and yield was clearly demonstrated. The highest morphological and yield-related parameters were observed in the PS-5 treatment, which utilized the deepest pot, indicating a direct correlation with enhanced root-system development. Under SB conditions, the use of small pots with limited volume can restrict root growth, potentially constraining overall plant development and preventing the full expression of the plants’ genetic potential. Therefore, determining the optimal pot size for SB applications is crucial. These findings suggest that integrating SB protocols with appropriate physical infrastructure components is essential for achieving more efficient and healthier plant growth.

Seedling establishment is a key challenge in wheat production, often assessed by plant numbers during early growth (Mahdi et al., 1998). Short coleoptiles can result in weak seedlings when sown deeply, as the first leaf struggles to emerge (Mahdi et al., 1998). In contrast, longer coleoptiles enhance the ability of plants to emerge from greater sizes (Schillinger et al., 1998; Rebetzke et al., 2007). Numerous studies have shown a positive correlation between coleoptile length and seedling emergence, particularly at greater sowing sizes (Schillinger et al., 1998; Takahashi et al, 1999; Matsui et al., 2002; Rebetzke et al., 2007; Sunderman, 1964).

**Figure 6:**
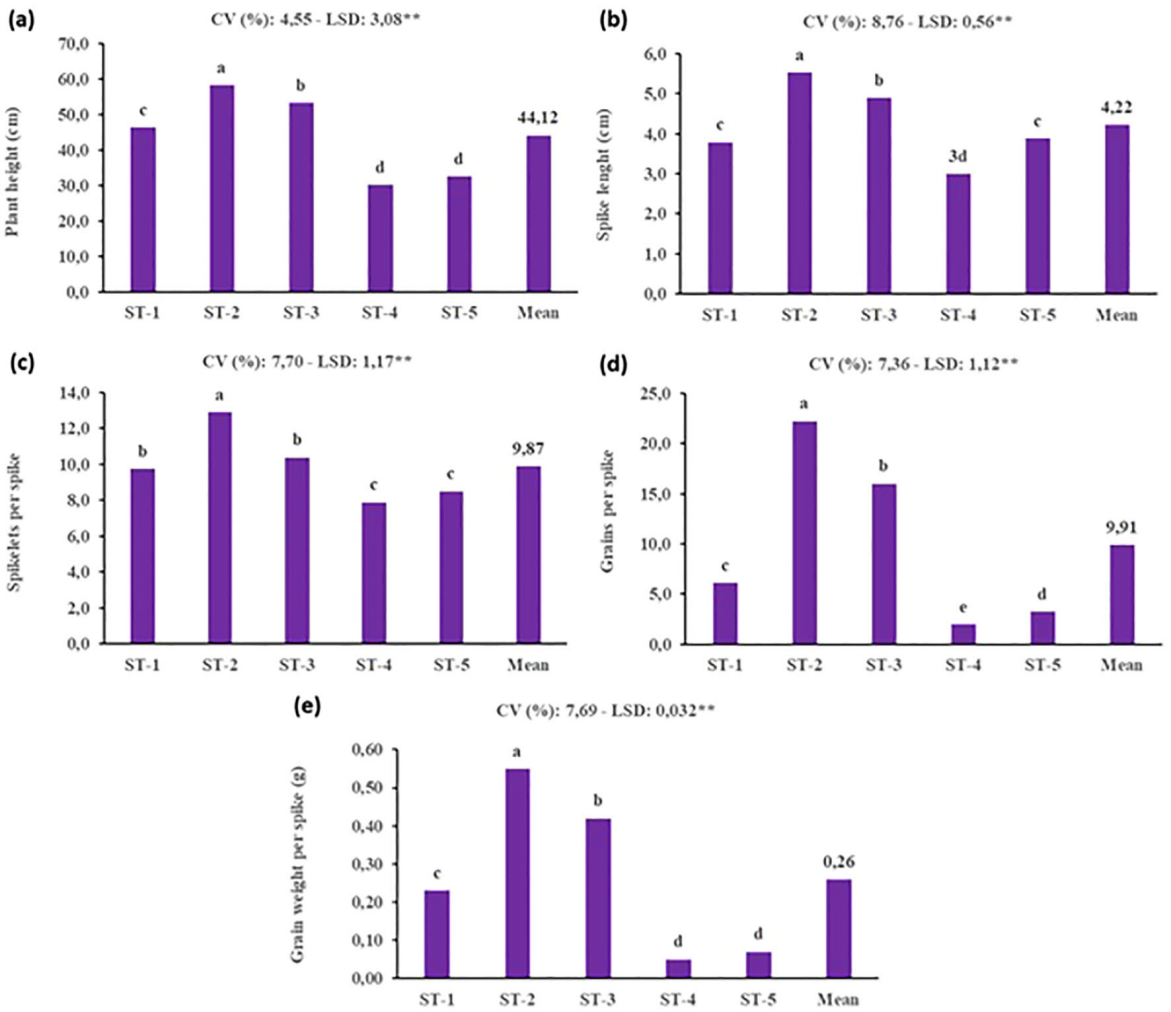
Effect of different soil types on yield and yield components of durum wheat under speed breeding condition (ST, Soil type, ST-1: %100 soil, ST-2: %100 peat, ST-3: %50 soil + %50 peat, ST-4: %40 soil + %30 peat + %30 farmyard manure, ST-5: %25 soil + %25 peat + %25 farmyard manure + %25 sand, CV, Coefficient of variation; LSD, Least significant difference). **(a)** plant height (cm), **(b)** spike lenght (cm), **(c)** spikelet per spike, **(d)** grains per spike and **(e)** grain weight per spike (g). The bar was presented as the standard deviation of means. Different lowercase letters indicated significant differences according to LSD’s test at the level of P<0.01.

Under SB conditions, the selection of soil type is crucial for optimizing plant growth and yield, given the accelerated life cycle and controlled environment. This study’s evaluation of various soil types and mixtures demonstrated that the ST-2 treatment, composed entirely of peat, yielded the highest morphological and yield-related values. Conversely, the poorest performance was observed in mixtures containing farmyard manure, likely due to the slower decomposition rate of organic matter, which can temporarily limit nutrient availability under rapid-growth conditions (**Figure 6**). The superior performance of peat can be attributed to its high-water retention capacity, aeration, and rich organic content—all of which are particularly advantageous in SB systems, where fast and uniform development is essential. Furthermore, peat facilitates deeper and healthier root growth, enhancing nutrient uptake efficiency and supporting vigorous shoot development. These findings underscore the importance of selecting a substrate that aligns with the physiological demands imposed by SB protocols. Unlike traditional cultivation systems, SB accelerates developmental stages, leaving little margin for delayed nutrient release or inconsistent root zone conditions. Thus, while farmyard manure may benefit long-term soil fertility in field conditions, its integration into SB media appears suboptimal. The application of vermicompost increased the height of plants due to present of essential nutrients (Praveesh et al., 2017). In addition, these researchers stated that in the experiment, the highest length of wheat plant was recorded in 50% soil treated with vermicompost as compaired to cattle dung treated soil and control soil.

**Figure 7:**
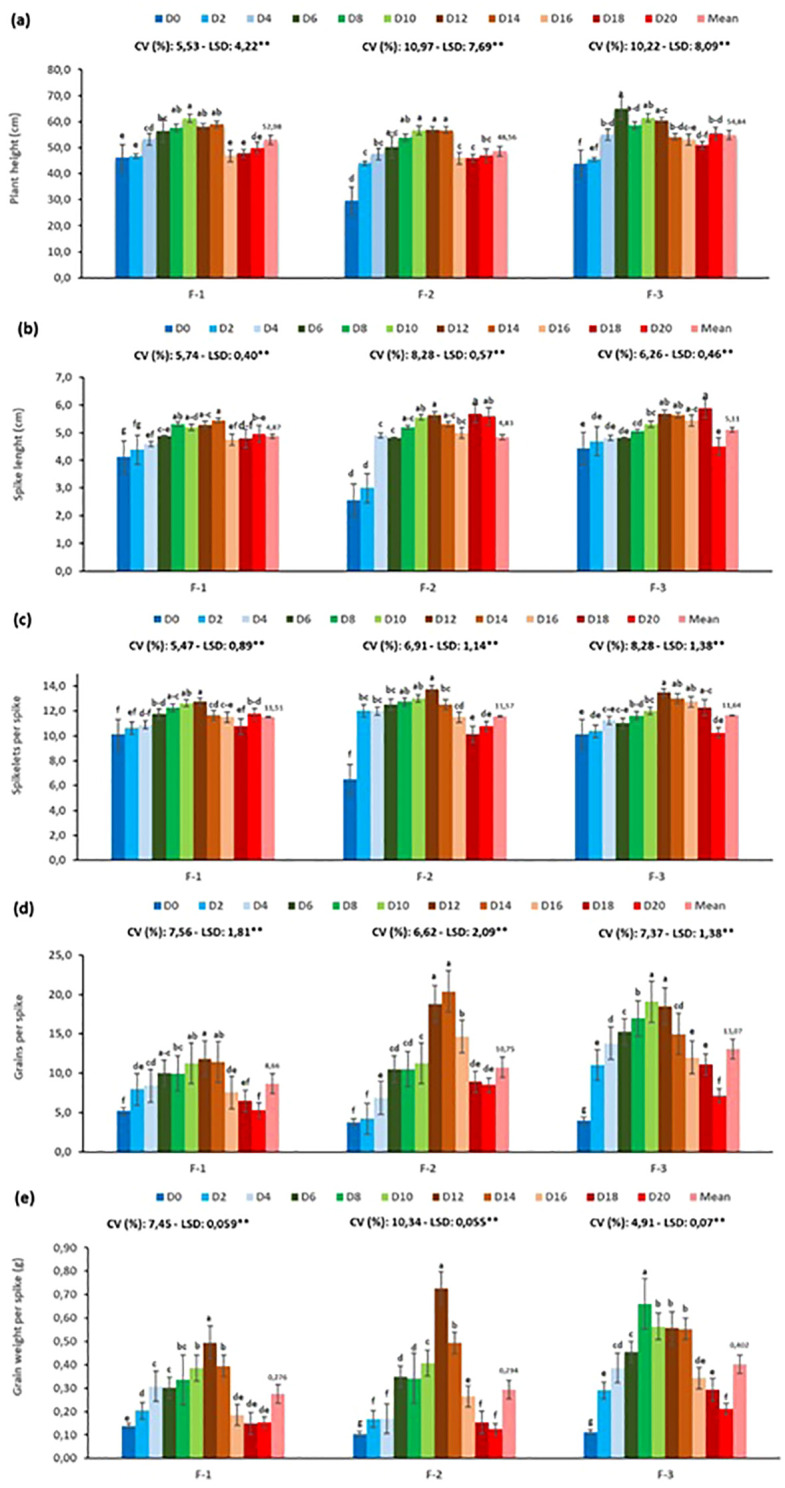
Effect of different fertilizer types and doses on yield and yield components of durum wheat under speed breeding condition (F, Fertilizer, F-1: CAN (%26), F-2: TSP (%43–44 P2O5) + CAN (%26), F-3: 20.20.0 (%20 N + %20 K), CV, Coefficient of variation; LSD, Least significant difference). **(a)** plant height (cm), **(b)** spike lenght (cm), **(c)** spikelet per spike, **(d)** grains per spike and **(e)** grain weight per spike (g). The bar was presented as the standard deviation of means. Different lowercase letters indicated significant differences according to LSD’s test at the level of P<0.01.

This study was conducted to assess the impact of various fertilizer types (CAN, TSP+CAN, and 20.20.0) and their respective dosages on the yield and yield components of durum wheat under SB conditions. The results clearly indicate that both the type and dosage of fertilizer significantly affect plant growth and yield-related parameters (**Figure 7**). For all traits examined—including plant height, spike length, number of spikelets per spike, number of grains per spike, and grain weight per spike—values increased with fertilizer dosage up to a certain threshold, beyond which a decline was observed. This suggests that while nutrients are beneficial up to an optimal level, excessive fertilization may negatively impact growth and productivity.

Among the three fertilizer types, the highest values were generally recorded in the F-3 (20.20.0) treatment. Containing 20% nitrogen and 20% phosphorus, this fertilizer effectively supported both vegetative growth and the development of roots and spikes. In the F-2 treatment (TSP + CAN), the high phosphorus content promoted root and spike formation; however, the lack of sufficient nitrogen limited yield performance compared to F-3. The F-1 treatment (CAN), providing only nitrogen, failed to compensate for phosphorus deficiency and consequently resulted in lower values across all measured traits. These findings underscore the positive impact of balanced fertilization strategies on plant growth and yield. Nonetheless, it is also important that fertilization practices are sustainable and cost-effective. As emphasized by Praveesh et al. (2017), the use of complementary, long-lasting nutrient sources that reduce reliance on chemical fertilizers is essential for sustainable crop production.

**Figure 8:**
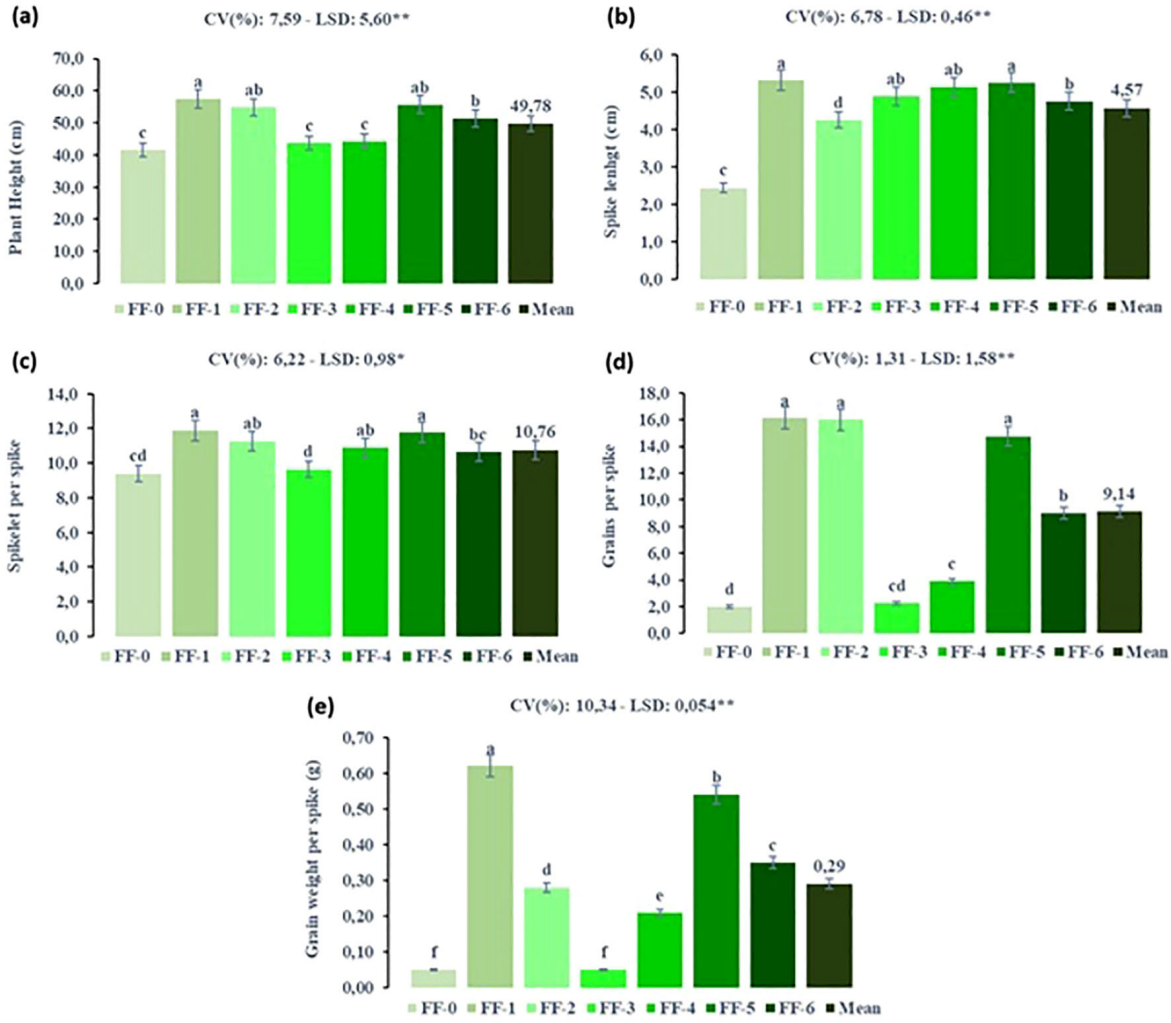
Effect of foliar fertilizer under speed breeding condition on yield and yield components of durum wheat at different growth stages (FF, Foliar fertilizer, FF-0: non foliar fertilizer, FF-1: tillering stage, FF-2: heading stage, FF-3: tillering-stem elongation, FF-4: tillering-heading, FF-5: stem elongation-heading stage and FF-6: tillering-stem elongation-heading stage, CV, Coefficient of variation; LSD, Least significant difference). **(a)** plant height (cm), **(b)** spike lenght (cm), **(c)** spikelet per spike, **(d)** grains per spike and **(e)** grain weight per spike (g). The bar was presented as the standard deviation of means. Different lowercase letters indicated significant differences according to LSD’s test at the level of P<0.01.

**Figure 8** illustrates the effects of foliar fertilizer application at different developmental stages on the growth and yield components of durum wheat under SB conditions. The results clearly demonstrated that the timing of foliar fertilizer application significantly influenced plant performance. The highest values for plant height, spike length, number of spikelets per spike, number of grains per spike, and grain weight per spike were generally recorded in the FF-1 (tillering stage) and FF-5 (stem elongation–heading stage) treatments. These findings emphasize the importance of applying foliar fertilizers at phenologically critical stages to optimize nutrient uptake and enhance the productivity.

The tillering stage (FF-1) is crucial for establishing the plant’s potential yield, as it is associated with the development of lateral shoots and roots. Foliar fertilizer application during this phase ensures rapid and efficient nutrient uptake, particularly under accelerated photoperiod and growth conditions typical of SB environments. Similarly, the FF-5 application, which coincides with the transition from stem elongation to heading, was highly effective. During this stage, wheat plants require substantial nutritional support for rapid vertical growth and reproductive development. Foliar fertilizer application at FF-5 enhanced stem strength and facilitated optimal spike formation and grain filling.

In contrast, treatments such as FF-2 (heading stage only) and FF-3 (tillering–stem elongation) resulted in lower trait values, suggesting that fertilization at these isolated or suboptimal timings may not sufficiently meet the dynamic nutritional demands of the plant during rapid developmental transitions in the SB settings. These findings are consistent with those of Wagan et al. (2017) and Khan et al. (2009), who reported that foliar application of urea, particularly during tillering and stem elongation stages, significantly contributed to yield enhancement in wheat.

In a similar study conducted to optimize SB conditions, Cha et al. (2023) reported that the results of the speed vernalization (SV) combined with the modified speed breeding (mSB) model, along with its practical application in wheat breeding programs, may assist breeders worldwide in integrating generation acceleration systems into conventional breeding programs. Bayhan et al. (2022) demonstrated that while reduced input conditions in a SB system led to significant declines in agronomic traits such as fertile tiller number, plant height, grain number per spike, and grain weight, the higher germination rate of seeds obtained under these stressful conditions suggests that low-input applications may still contribute positively to the success of SB programs.

## 4 Conclusion

This study examined the effects of pot size, soil type, fertilizer, and foliar fertilizer applications on wheat growth under SB conditions. The highest productivity was observed with the deep pot (PS-5), 100% peat soil (ST-2), and F-3 fertilizer application. Among the foliar fertilizer applications, the best results were achieved during the tillering and between stem elongation-heading stage stages. In all fertilizer applications, an increase in values was observed up to a certain dose, followed by a subsequent decrease. These findings provide insights into optimizing plant productivity and ensuring an efficient breeding process under SB conditions. As the main goal of SB is to obtain multiple generations in a short time, it is crucial to determine the most suitable pot size, soil mixture, fertilizer, and foliar applications that support plant growth and development. These results highlight the key factors to make the SB process more efficient and cost-effective. By accelerating plant growth and shortening generation times, these practices will contribute to the faster development of more resilient and productive plant varieties in the future.

## Data Availability

The original contributions presented in the study are included in the article/supplementary material. Further inquiries can be directed to the corresponding author.
